# No Association Between Hypnotizability and Basal Ganglia Morphometry

**DOI:** 10.3390/brainsci16030287

**Published:** 2026-03-04

**Authors:** Eleonora Picerni, Fabrizio Piras, Daniela Laricchiuta, Debora Cutuli, Laura Petrosini, Enrica Laura Santarcangelo

**Affiliations:** 1 IRCCS Santa Lucia Foundation, 00179 Rome, Italy; eleonora.picerni@gmail.com (E.P.); f.piras@hsantalucia.it (F.P.); debora.cutuli@uniroma1.it (D.C.); laura.petrosini@uniroma1.it (L.P.); 2 Department of Philosophy, Social Sciences & Education, University of Perugia, 06123 Perugia, Italy; daniela.laricchiuta@uniroma1.it; 3 Department of Psychology, Sapienza University, 00185 Rome, Italy; 4 Department of Translational Research and New Technologies in Medicine and Surgery, University of Pisa, 56127 Pisa, Italy; 5 Istituto di Fisiologia, Via San Zeno 31, 56127 Pisa, Italy

**Keywords:** basal ganglia, hypnotizability, Stanford Hypnotic Susceptibility Scale, Voxel-Based Morphometry, individual differences

## Abstract

**Highlights:**

**What are the main findings?**

- Hypnotizability does not display basal ganglia (BG) structural variations.
- This is at variance with the reported variations in cerebellum and insula.

**What are the implications of the main findings?**

- BG do not contribute to the insula/cerebellum-related behavioral differences.
- Negative findings clarify hypnotizability-related physiological mechanisms.

**Abstract:**

**Background:** Hypnotizability is a psychophysiological trait measured by standardized scales whose scores indicate the proneness to modify perception, memory, and behavior according to specific suggestions, and to experience the hypnotic state. Hypnotizability exhibits physiological and behavioral correlates in the ordinary state of consciousness and brain morphological peculiarities. Among them, a reduced Grey Matter volume (GMV) of the insula and cerebellar lobules IV–VI has been observed in high hypnotizable individuals (highs) compared to low hypnotizables (lows). Theoretically, these structures could cooperate with the basal ganglia (BG) in hypnotizability-related sensorimotor and cognitive–emotional differences. **Methods:** Since no imaging study has focused on the BG as a function of hypnotizability, the present research investigated the morphometric characteristics of the BG in the highs (N = 12) and lows (N = 37) enrolled in a previous study focused on the cerebellum. **Results:** The results did not show any significant difference in the GMV of the caudate and pallidum. **Conclusions:** Thus, the hypnotizability-related behavioral and physiological difference, as well as the responsiveness to hypnotic suggestions associated with the insula and cerebellum variations, are not partially accounted for by BG structural differences.

## 1. Introduction

Hypnotic susceptibility (hypnotizability) is a stable individual trait enabling a few individuals to experience the hypnotic state and to modify perception, memory, and behavior according to the content of specific suggestions—i.e., requests to imagine and perceive personal and environmental contexts different from the actual ones—in both hypnosis and the ordinary state of consciousness [1,2]. Hypnotizability is measured by standard scales, which classify the general population into high (highs), medium (mediums), and low (lows) hypnotizable individuals [3].

Many lines of evidence suggest that hypnotizability is a hierarchically structured cognitive capacity, rooted in multiple sub-abilities (attentive skills, imagery vividness, fantasy proneness) that interact to give rise to individual differences in hypnotizability [4]. Hypnotizability levels also show distinct physiological correlates observable in the ordinary state of consciousness and in the absence of suggestions. They regard the highs’ greater stability of attention, stronger functional equivalence between imagery and perception/action and information processing, higher motor cortex excitability, less accurate sensorimotor integration, low accuracy in the detection of visceral signals and their more adaptive interpretation (defined sensitivity), more favorable cardiovascular regulation [5], and different emotional control [6,7].

Brain structural variations can account for individual differences in physiological and behavioral differences independently from functional assessment [8].

Hypnotizability-related differences include brain morphological peculiarities, such as a reduced total brain volume [9] and Grey Matter (GM) volume of the insula and cerebellum [10,11] in highs compared to lows. Both the insula and the cerebellum are integrated into neural networks including the Basal Ganglia (BG) and participate in the default, salience, and executive systems [12]. Several studies have evidenced that these two structures cooperate in sensorimotor control and reward-driven behavior, in the processing of information with a motivational valence, and in the neural substrate of some personality traits [13,14].

The insula structural characteristics can be involved in the highs’ low interoceptive accuracy and influence their cognitive–emotional control [7]. The highs’ reduced GM volumes in the left cerebellar lobules IV–VI, described in a previous study [11], have been considered responsible for the highs’ less close postural and visuo-motor control with the absence of learning across consecutive trials and for the greater excitability of the right motor cortex in resting conditions [5]. Notably, the paradoxical control of experimental pain induced by bilateral cerebellar transcranial Direct Current Stimulation (tDCS), consisting of increased amplitude of the nociceptive brain potential and reported pain intensity, can also be ascribed to an anomalous interaction between the cerebellum and the pain matrix [15].

Anatomically, the subthalamic nucleus of the BG has a disynaptic projection to the cerebellar cortex [16,17], and the cerebellar dentate nucleus has a disynaptic projection to the striatum [18]. Moreover, cerebellar direct projections contact the substantia nigra [19] and the internal pallidum [16,17]. A recent study highlights that the BG and cerebellum collaborate in a performance-related functional network that processes performance outcomes, as shown by the cerebellar inhibition by the ventral striatum following successful motor outcomes, and by the modulation of their relation by motivation and subjective preferences [20]. Moreover, the BG cooperate with the insula [21] and cerebellum within a network controlled by the prefrontal cortex [7,22], possibly influencing the cognitive–emotional control.

To date, no neuroimaging study has focused on BG GMV as a function of hypnotizability, although they contribute to complex motor, cognitive, and emotional behaviors, cooperating with other motor and non-motor structures, including the cerebellum and the insula [17,23].

Thus, under the hypothesis that the BG participate in an integrated hypnotizability-related network involving cerebellum and insula, the present research aimed at exploring eventual structural variations of the BG in highs and lows.

## 2. Methods

### 2.1. Participants

The study analyzed brain images from 49 healthy, right-handed individuals (23 males, mean age 40.38 ± 11.83 years) who participated in prior research on cerebellar involvement in the hypnotizability trait [11]. A new ethics review and informed consent were not needed because the current study was a retrospective analysis of existing data (local Ethics Committee approval n. CE/PROG.461 (September 22nd, 2014).

All participants were right-handed (Edinburgh Handedness Inventory [24]) and MRI-compatible. Exclusionary criteria were cognitive impairment or dementia assessed by Mini-Mental State Examination [25]; Mental Deterioration Battery [26] and NINCDS-ADRDA criteria for dementia [27]; subjective reports of any cognitive deficit; and the diagnosis of major systemic medical, neurological, and psychiatric disease. Moreover, a trained neuroradiologist and a neuropsychologist co-inspected the acquired MRI sequences (i.e., T1- and T2-weighted and FLAIR images) to exclude structural abnormalities (i.e., FLAIR or T2-weighted hyperintensities and T1-weighted hypointensities).

#### Hypnotic Assessment

Hypnotic assessment was conducted in healthy volunteers through the Italian version of the Stanford Hypnotic Susceptibility Scale, Form A (SHSS: A, [28]), which consists of 12 items exploring various aspects of hypnotizability, i.e., motor inhibition, tendency to delusions, and dissociation. It classifies the general population as high (“highs”, SHSS core ≥ 8) or low hypnotizable (“lows”, SHSS score ≤ 4). Each group represents about 15% of the population, which is mainly represented by mediums (70%) [3]. In line with previous studies [29,30], and to align the present analyses with those performed on the MRI images acquired earlier from the same participants [11], mediums (SHSS score 5–7) were excluded. The enrolled group consisted of 12 highs (4 males; SHSS: A score: (mean ± SD) 9.4 ± 1.3; age: 41.25 ± 12.60 years; education: 15.58 ± 2.64 years) and 37 lows (19 males; SHSS-A score: 1.1 ± 1.5; age: 40.10 ± 11.74 years, range: 23-62 years; education: 16.02 ± 3.19 years).

### 2.2. Image Acquisition

All participants underwent a comprehensive MRI scan protocol performed on a 3-Tesla Allegra MR Imager (Siemens, Erlangen, Germany). The imaging protocol comprised standard clinical sequences, namely a dual-echo turbo spin echo (TSE) and a T2 fluid attenuated inversion recovery (FLAIR) for conventional MRI visualization of the brain and exclude gross brain abnormalities. High-resolution structural imaging was obtained using a volumetric whole-brain three-dimensional T1-weighted sequence acquired in the sagittal plane with a modified driven equilibrium Fourier transform (MDEFT) sequence (echo time/repetition time (TE/TR) = 2.4/7.92 ms, flip angle 15°, voxel size 1 × 1 × 1 mm^3^). This sequence provided optimal contrast for voxel-based morphometric analyses of GM and White Matter (WM) volumes. All planar acquisitions were aligned parallel to the anterior–posterior commissure (AC-PC) line. Special care was taken to center participants’ heads within the head coil to minimize magnetic field inhomogeneities and susceptibility artifacts.

### 2.3. Image Processing

To explore the link between regional brain volumes (of the BG) and hypnotic susceptibility, we performed a voxel-by-voxel analysis. The T1-weighted images were processed using the CAT12 toolbox (C. Gaser, Structural Brain Mapping group, Jena University Hospital, Jena, Germany) of SPM12, running in Matlab R2016a (MathWorks, Natick, MA, USA). CAT12 extends the unified segmentation framework [31] by integrating bias field correction, tissue classification, and spatial normalization in a unified preprocessing pipeline, optimized to enhance data quality and anatomical precision. Initially, an optimized block-wise non-local means denoising filter with noise adaptation [32] was applied to increase the signal-to-noise ratio of the structural images. Subsequently, an adaptive maximum a posteriori segmentation algorithm, incorporating partial volume estimation, was used to classify images into GM, WM, and cerebrospinal fluid (CSF). This step was followed by the application of a hidden Markov random field model, which imposed spatial constraints on tissue probability maps, reducing the likelihood of isolated misclassified voxels and improving anatomical coherence by filling holes within tissue clusters. High-dimensional spatial normalization was then performed using the Diffeomorphic Anatomical Registration Through Exponentiated Lie Algebra (DARTEL) algorithm [33]. This iterative approach allowed accurate registration of individual tissue maps to the stereotaxic space of the Montreal Neurological Institute (MNI). The resulting deformation fields were applied to modulate GM and WM maps, preserving local volumetric information after normalization. Voxel values in the modulated images thus reflected the relative contribution of each voxel to the total tissue volume. Following normalization, GM and WM maps were resampled to an isotropic voxel size of 1.5 mm^3^ and smoothed with an 8 mm full-width at half-maximum (FWHM) Gaussian kernel, in accordance with standard Voxel-Based Morphometry (VBM) procedures. Visual inspection of all images was conducted to exclude preprocessing artifacts, and a quantitative quality control assessment was performed using CAT12 to ensure homogeneity of GM segmentation across participants. The resulting processed GM images were used for BG volumetric analyses.

### 2.4. Basal Ganglia Masking

A key part of the process was creating Region-Of-Interest (ROI) masks for the BG, which included the caudate, putamen, and pallidum. These masks were created by meaning VBM-based GM probability maps obtained in the processing steps, thresholding the relative image to a value of 0.3 (i.e., removing all voxels having a probability to belong to GM or WM lower or equal to 29%). The threshold of 0.3 was chosen to ensure that only voxels with a high probability of being BG were included in the ROI, effectively minimizing partial volume effects from surrounding WM or CSF, which is a standard procedure in VBM of subcortical structures.

Then, all the non-BG structures were manually removed using the MNI-oriented atlas of the human brain (Automated Anatomical Labeling Atlas, AAL3) [34]. Two experienced researchers (F.P., E.P.) independently performed the manual removal of non-BG structures. Crucially, the researchers were blinded to the hypnotizability level of each participant during the entire masking process to prevent any observer bias. The high level of agreement between the two researchers (inter-rater reliability) further validated the reproducibility of the final masks.

The substantia nigra was not included in the analyses due to methodological constraints, as the MRI protocol did not include sequences (e.g., T2* or susceptibility-weighted imaging) required for its reliable segmentation and no validated automated algorithms were available.

### 2.5. Statistical Analysis

Our sample size was comparable to other studies’ investigating multiple brain regions in two groups [35].

A chi-squared (χ^2^) test was used for sex, while unpaired *t*-tests were used for age and education. The significance level for demographic characteristics was set at *p* < 0.05.

To analyze the differences in BG volumes, *t*-tests between highs and lows were singularly performed (one for each BG) at the voxel level using the CAT12 toolbox within the framework of the General Linear Model (GLM). Total Intracranial Volume (TIV), age, and years of education were included as covariates to control for their potential influence on the results. Sex was also used as a dummy variable, due to its dichotomic nature. To manage the risk of false positives, we applied a Family Wise Error (FWE) correction and considered results belonging to contiguous clusters of at least 20 voxels. Given the small volume of BG, the study additionally considered significant results obtained at a more lenient uncorrected threshold of *p* < 0.001. After the analysis, the mean BG values were extracted for both groups, and histograms were created to visualize the findings. Mean extracted volumes were also used to compute the traditional frequentist statistics (Student’s *t*-tests).

To quantify the evidence in favor of the null hypothesis (i.e., structural equivalence between groups), we complemented the traditional frequentist analyses with Bayesian Independent Samples *t*-tests on extracted mean GM volumes of each BG nucleus using the JSQ module running in Jamovi (Version 2.3, retrieved from https://www.jamovi.org). For each nucleus, we calculated the Bayes Factor (BF_01_), which represents the likelihood of the data under the null hypothesis (H_0_) relative to the alternative hypothesis (H_1_). A default Cauchy prior with a scale of 0.707 was employed. To further validate the reliability of our null findings, we performed a Bayes Factor Robustness Check for each region. This analysis evaluates how the evidence for the null hypothesis (H_0_) changes when different prior distributions are assumed, assessing the stability of the results.

## 3. Results

We did not observe a significant effect of sex in both lows (χ^2^ = 0.03; d.f. = 1; *p* = 0.87) and highs (χ^2^ = 1.33; d.f. = 1; *p* = 0.25). Highs and lows did not differ significantly in age (t = 0.28; d.f. = 47; *p* = 0.77) and educational level (t = −0.43; d.f. = 47; *p* = 0.66).

Student’s *t*-tests on the BG extracted volumes yielded non-significant results for the caudate (*p* = 0.712), putamen (*p* = 0.405), and pallidum (*p* = 0.601); negligible Cohen’s d effect sizes (ranging from 0.015 to −0.078); and the 95% Credibility Intervals widely overlap with zero. Table S1 (Supplementary Materials) summarizes the results.

**Figure 1:**
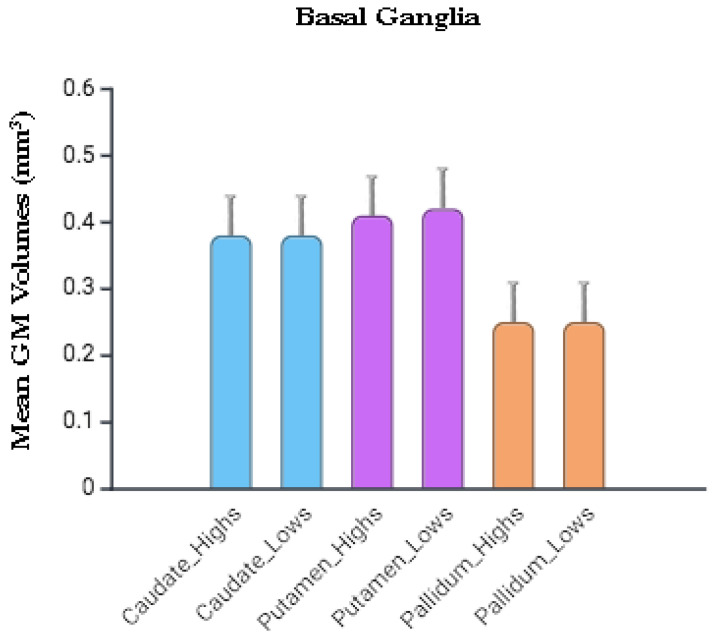
Basal ganglia Grey Matter volume. Basal ganglia GMV (Mean, SEM) of highs and lows. Histograms were created in Biorender (https://BioRender.com).

The Bayesian analysis on the extracted volumes (Figure 1, Supplementary Materials) yielded moderate evidence in favor of the null hypothesis for all examined regions (caudate: BF_01_ = 3.28; pallidum: BF_01_ = 3.05; putamen: BF_01_ = 3.33). Visual inspection of the Prior-and-Posterior plots revealed that for each nucleus, the posterior density at an effect size of zero was notably higher than the prior density, confirming that the data shifted our belief toward the absence of structural differences. Furthermore, the Robustness Check indicated that the support for the null hypothesis remained stable or increased as the prior width was widened (in the caudate, the BF_01_ reached values up to 5.26 for the caudate under an ‘ultrawide’ prior; in the putamen, the BF_01_ reached 4.044; in the pallidum, the BF_01_ increased to 4.926).

## 4. Discussion

The present study was prompted by the anatomical and functional relationships between BG and the earlier investigated cerebellum and insula, whose hypnotizability-related structural variations have been associated with behavioral and physiological differences between highs and lows. The findings suggest that BG do not contribute to the physiological and behavioral differences associated with the cerebellum’ and insula’ GMV variations, as their volumetry does not vary as a function of hypnotizability levels.

The absence of significant volumetric differences in the BG was supported by Bayesian statistics. Specifically, the BF_01_ values were consistently above 3 for the caudate, pallidum, and putamen, suggesting that the null results in the VBM are not due to a lack of statistical power but rather reflect a genuine structural similarity between the groups. By demonstrating that the data are more than three times more likely under the null hypothesis than the alternative, we can conclude with moderate confidence that hypnotizability does not impact the macro-structural morphometry of the BG in our sample. Furthermore, the evidence favoring structural equivalence between highs and lows is robust and not sensitive to the specific choice of prior. Indeed, even when allowing for the possibility of larger effect sizes (Bayes Factor Robustness Check), the data consistently point toward an absence of volumetric differences between the two groups.

### 4.1. Sensorimotor Domain

The differences observed in sensorimotor integration—less close postural and visuomotor control in highs than in lows, and the absence of learning across trials—are well explained by the cerebellum morpho-functional differences [5] even without any BG contribution. To date, other sensorimotor differences possibly related to impairment of the BG functioning, such as slow voluntary movement, low amplitude and speed of repetitive movements, and delayed movement initiation, have not been reported as a function of hypnotizability. The latency of suggested movements, anecdotally observed in highs and more pronounced after hypnotic induction, can be due to a delay in the shift of attention from the internal world to the object of the received suggestions. Indeed, highs are known to display greater stability of attention than lows, which has been attributed to greater dopamine concentration in their prefrontal cortex. Nonetheless, the evidence about the association of higher cortical dopamine content—based on the higher frequency of the ValMet polymorphism of the Catechol-O-Methyl-Transferase (COMT) associated with reduced dopamine catabolism—and higher attentional stability in highs is not consistent [36]. The inertia in starting movement could be due to the relaxation [37] associated with the classical hypnotic induction, which is demonstrated by the parasympathetic prevalence in the control of heart rate [5], and with a reduced sense of agency [38]. Finally, both relaxation and neutral hypnosis display progressively reduced spinal excitability for lower limbs and left upper limbs [5].

### 4.2. Cognitive–Emotional Domain

Cognitive–emotional alterations can be accounted for by both the cerebellum and BG’s altered functioning, as they participate in the same neural networks [12]. In the general population, low-frequency cerebellar repetitive Transcranial Magnetic Stimulation (TMS) impairs emotional regulation, and high-frequency cerebellar repetitive TMS improves the processing of positive emotional stimuli [39]. Facilitation of the recognition of negative emotional faces has also been observed following cerebellar tDCS [40,41].

The BG are involved in motivation, reward learning, habit formation, and emotional regulation owing to their connections with the prefrontal cortex and limbic structures. Their functional impairment causes delayed action initiation, slowed thinking, reduced cognitive flexibility, mood alterations, and impulse control difficulties.

Beyond the suggested greater cortical dopamine content [36], the highs’ difficulty in shifting attention could be ascribed to both the cerebellum and the BG’s altered functioning. Nonetheless, highs do not exhibit further cognitive symptoms possibly ascribable to these structures [42], although a higher percentage of anxiety disorders has been observed among highs [43]. The absence of differences between highs and lows in the BG GMV fits with the lack of hypnotizability-related differences in the tendency to approach novelty and reward, assessed through questionnaires exploring the activity of the Behavioral Inhibition/Activation System [5]. In contrast, highs differ from lows in the relationship between risk perception, risk-taking, and expected benefit, as highs take their decisions based on the perceived risk, whereas lows base them on the expected monetary benefit [6].

Emotion is at least partially accounted for by interoception, that is, the ability to detect (accuracy) and interpret (sensitivity) visceral information. Thus, emotional differences between highs and lows can be more likely accounted for by the differences observed in the insula, which is the main hub for interoception. Its weaker functional connectivity with the cerebellum has been linked to higher emotional susceptibility [18,44]. Notably, the highs’ higher emotionality and empathy may also derive from their stronger involvement in actual and imagined experiences [5]—measured by the Tellegen Absorption Scale [45]—which could secondarily induce greater engagement of emotional circuits.

Intriguingly, it has been recently reported that the cerebellum modulates the dopamine levels in the BG, conveying information related to both movement and reward [19]. Thus, we may suggest that, beyond the high dopamine concentration in the highs’ cerebral cortex, the highs’ cortical dopaminergic activity could be related to reduced cerebellar inhibition on the substantia nigra and internal pallidum. Unfortunately, the substantia nigra [19] could not be studied through our MRI protocol.

We are fully aware that other structural differences—i.e., the highs’ greater GMV in the medial and superior frontal gyrus, mid-temporal and mid-occipital cortices, greater white matter volume in the corpus callosum)—and functional characteristics—i.e., the highs’ stronger functional connection between the anterior cingulate and the dorsolateral prefrontal cortices—could participate in the reported behavioral and physiological differences between highs and lows which have been associated with structural differences in the insula and cerebellum [5]. Moreover, a sample reflecting the distribution of hypnotizability in the general population [3], along with a more sophisticated classification of hypnotizability considering different types of highly hypnotizable individuals, may provide more precise results [46]. Indeed, Stanford Scales include at least three factors, i.e., the proneness to motor inhibition, hallucination, and dissociation, and the same score can be obtained passing different items. The hypothesis advanced regarding the reduced GMV in the insula and cerebellum refers to the high nitric oxide content of these brain regions [5] Indeed, nitric oxide is necessary to neuronal maturation, but its excessive amount is detrimental to the nervous tissue [47], particularly in the regions with large amounts of nitric oxide. Nonetheless, this hypothesis must be confirmed by experimental studies

## 5. Conclusions

We did not detect any volumetric differences in the BG, in contrast to the highs’ reduced volume of the insula and left cerebellum observed in the earlier study conducted in the same sample [11] and in other studies [10]. Bayesian analyses (Bayes Factor Robustness Check) revealed that investigations in a larger sample could not reveal further differences. Thus, despite the relations of BG with the cerebellum and insula [16,17,18], they do not contribute to the behavioral and physiological differences observed between highs and lows.

Finally, we wish to remark that negative findings can be as relevant as positive findings are. In the present study, they provide deeper insight into the morphological brain characteristics of individuals with different hypnotizability levels and suggest a more comprehensive interpretation of a few hypnotizability-related behavioral and physiological characteristics.

## Data Availability

The data presented in this study are available on request from the corresponding author due to their inclusion in the IRCCS Santa Lucia private repository.

## Supplementary Materials

The following supporting information can be downloaded at: https://www.mdpi.com/article/10.3390/brainsci16030287/s1, Table S1: Summary of Bayesian Independent Samples *T*-test results for Basal Ganglia; Figure S1: Bayesian analyses.
