# Supplementary material for: No Association Between Hypnotizability and Basal Ganglia Morphometry
Source: Brain Sci. 2026 Mar 4;16(3):287. doi: 10.3390/brainsci16030287 (PMC13024687; doi:10.3390/brainsci16030287)
Supplement: Supplementary file 1 [file brainsci-16-00287-s001.zip › brainsci-4162576-supplementary.pdf]

Table S1. Summary of Bayesian Independent Samples T-test results for Basal Ganglia.

**Regionof Interest (ROI)**	**Student's t**	**gdl**	**Exact *p* value**	**Cohen's d**	**BF_{01}**	**95% Credibility Interval**
**Caudate**	-0.371	47	**0.712**	0.015	**3.282**	(-0.483, 0.521)
**Putamen**	-0.840	47	**0.405**	-0.050	**3.328**	(-0.585, 0.483)
**Pallidum**	-0.527	47	**0.601**	-0.078	**3.046**	(-0.576, 0.531)

BF_{01} indicates the Bayes Factor value in favor of the null hypothesis.
Prior width was set to a default Cauchy scale of 0.707.

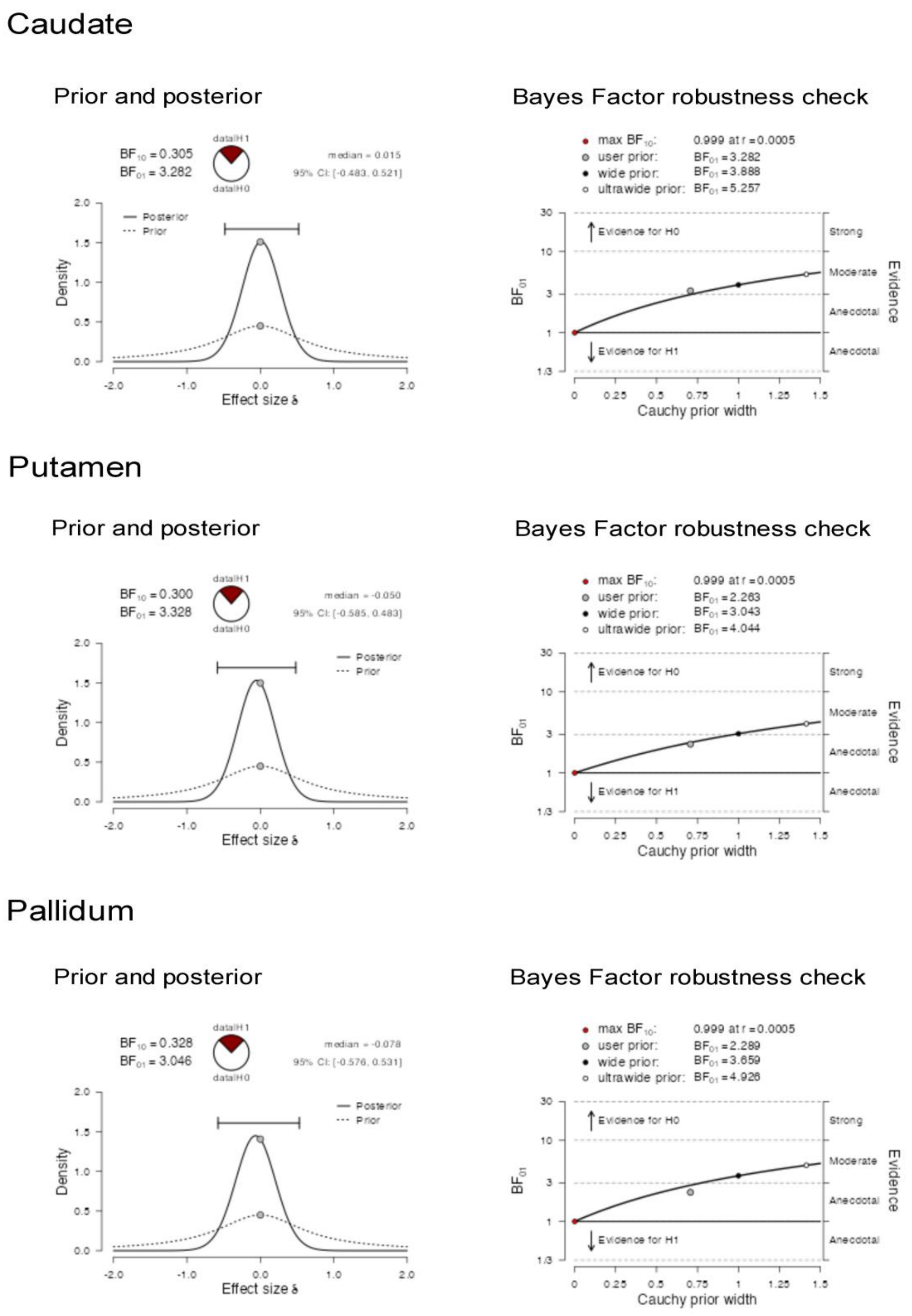

Figure S1. Bayesian analyses.
