# Supplementary figures and images for: No Association Between Hypnotizability and Basal Ganglia Morphometry
Source: Brain Sci. 2026 Mar 4;16(3):287. doi: 10.3390/brainsci16030287 (PMC13024687; doi:10.3390/brainsci16030287)

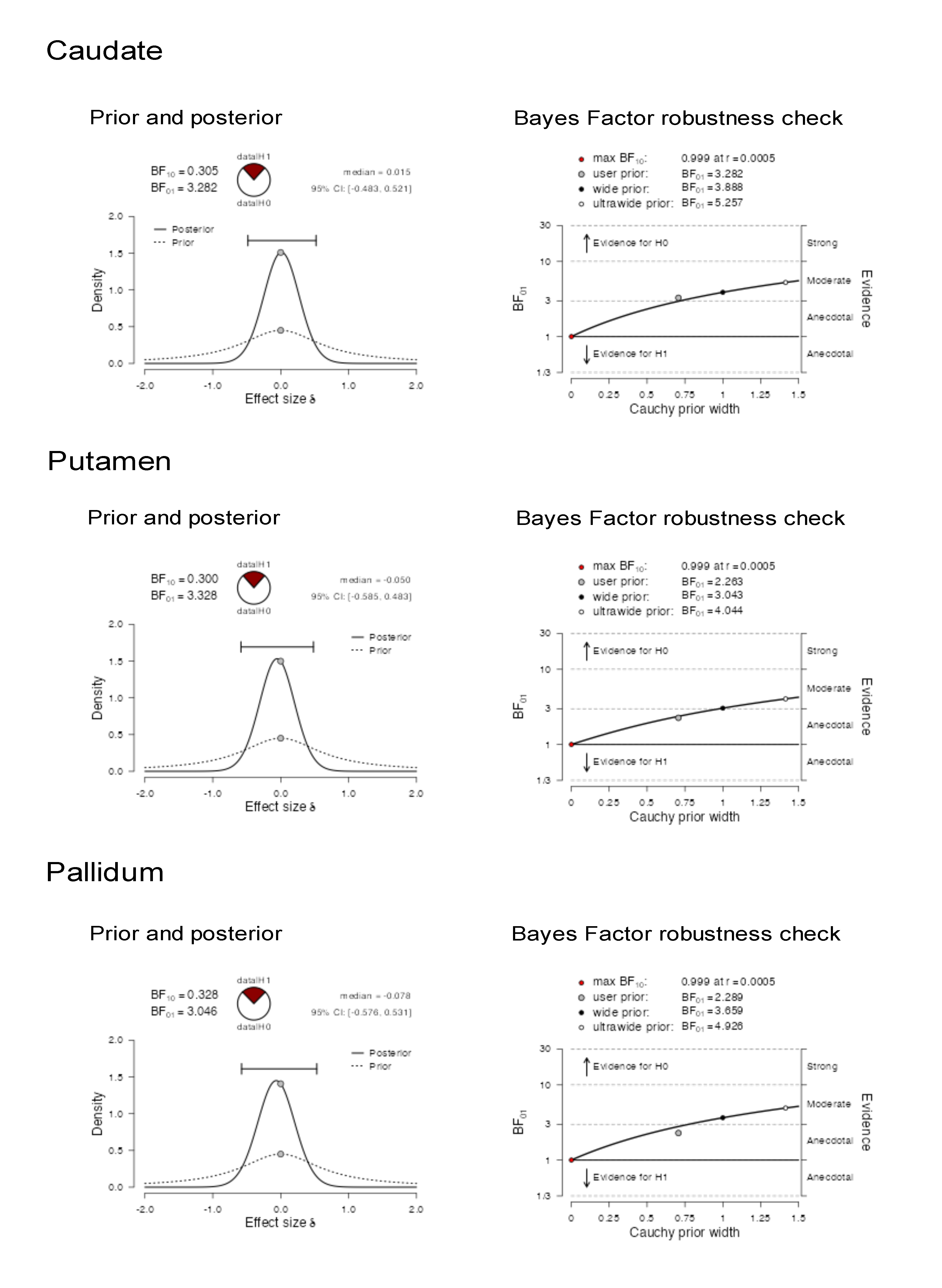

Supplement: Supplementary file 1 [file brainsci-16-00287-s001.zip › brainsci-4162576-Figure S1.tif]
